# P74 - Brittle asthma: a clinical phenotype of severe asthma

**DOI:** 10.1186/2045-7022-4-S1-P129

**Published:** 2014-02-28

**Authors:** Maria Zoto, Albana Deliu, Elida Nikolli, Mehmet Hoxha, Alfred Priftanji

**Affiliations:** 1 University Hospital Centre, Tirana, Albania

## Background

The term Brittle Asthma was generally used to describe those patients with sudden, severe attacks, usually out of the blue. Recently the following classification was suggested: type 1 characterized by a maintained wide PEF variability despite medical therapy, and type 2 characterized by sudden acute attacks on a background of apparently normal airway function. Patients who develop sudden-onset near-fatal asthma seem to have massive allergen exposure and emotional distress. Diminished perception of dyspnea and reduced compliance with anti-inflammatory therapy have been associated with fatal or near-fatal events.

## Case

We are presenting two case reports of two patients admitted to emergency service due to severe asthma attacks.

The first, 31 year-old patient with clinical history: allergic rhinoconjunctivitis since the age of 16 and untreated atopic asthma since the age of 20. At the moment of hospitalization he was without conscience, with tachypnea, cyanosis and the following vital signs: temperature 37.4°C, blood pressure 90/60 mmHg, pulse rate 140 bpm.

The second, 40 year-old patient, was admitted with cyanosis, breathless and agitated with the following vital signs: temperature 37°C, blood pressure 70/40 mmHg, pulse rate 130 bpm. ABG yielded the following: PO2 87.8 mmHg, PCO2 70.9 mmHg, pH 7.14, BE 8.4. His clinical history was: allergic asthma and rhinoconjunctivitis since the childhood, reappearance of asthma at the age of 22, and frequent avoidance of steroid therapy.

At the thoracic examination diffuse inspiratory and expiratory wheezes with prolonged expiratoy phase were present. Chest radiography and EKG were normal. Laboratory: blood count, renal, liver functions were normal. They were successfully resuscitated and recovered completely after aggressive treatment.

## Conclusions

We considered our patients as type 2 brittle asthma. Poor adherence with steroid treatment, ignoring the symptoms of airways obstruction, and emotional distress are offending factors for these near fatal attacks. Patients with brittle asthma are difficult to manage. They have to be monitored firmly and treat the acute attacks with self-administered subcutaneous adrenaline.

